# Post exposition to etoricoxib in patients with negative oral drug provocation tests

**DOI:** 10.1186/1939-4551-8-S1-A186

**Published:** 2015-04-08

**Authors:** Thais Sterza, Pedro HR Fernandes, Cristiane Momoi, Luis Felipe Ensina, Inês Cristina Camelo Nunes, Dirceu Solé

**Affiliations:** 1 Emescam College of Health Sciences, Brazil; 2 Federal University of São Paulo, Brazil

## Background

The etoricoxib can be used as a therapeutical alternative to patients with hypersensitivity to non-steroidal anti-inflammatory drugs (NSAIDs). The aim of this study was to evaluate the use of etoricoxib in daily life of patients with negative oral drug provocation tests (DPT).

## Methods

Patients with hypersensitivity to NSAIDs that performed a DPT with etoricoxibe between january/2013 and june/2014 were contacted by phone and asked about posterior etoricoxib drug usage and the occurrence of reactions. Those who had not used again were asked the reasons for not using.

## Results

Forty-one patients were tested, all with negative results. Thirty seven of them (90%) were contacted and 28 (76%) remembered the tested drug’s name. Twelve of them (32%) had used the drug again without any reaction. Twenty five patients were not re-exposed: 18 did not need, 4 were afraid of a new reaction and 3 used another alternative. Of the 4 patients who were afraid to use the drug again, 3 of them had presented anaphylaxis symptoms with other NSAIDs.

## Conclusions

Most of the patients exposed to etoricoxib DPT haven’t been exposed again to the drug. Of those who were afraid to use the drug again, most had a severe reaction history to NSAIDs.

